# Contrast-Enhanced Microtomographic Characterisation of Vessels in Native Bone and Engineered Vascularised Grafts Using Ink-Gelatin Perfusion and Phosphotungstic Acid

**DOI:** 10.1155/2017/4035160

**Published:** 2017-04-23

**Authors:** Sarah Sutter, Atanas Todorov, Tarek Ismail, Alexander Haumer, Ilario Fulco, Georg Schulz, Arnaud Scherberich, Alexandre Kaempfen, Ivan Martin, Dirk J. Schaefer

**Affiliations:** ^1^Department of Plastic, Reconstructive, Aesthetic and Hand Surgery, University Hospital Basel, University of Basel, Spitalstrasse 21, 4031 Basel, Switzerland; ^2^Department of Biomedicine, University Hospital Basel, University of Basel, Hebelstrasse 20, 4031 Basel, Switzerland; ^3^Department of Biomedical Engineering, University of Basel, Gewerbestrasse 14, 4123 Allschwil, Switzerland

## Abstract

**Objectives:**

Bone ischemia and necrosis are challenging to treat, requiring investigation of native and engineered bone revascularisation processes through advanced imaging techniques. This study demonstrates an experimental two-step method for precise bone and vessel analysis in native bones or vascularised bone grafts using X-ray microtomography (*μ*CT), without interfering with further histological processing.

**Methods:**

Distally ligated epigastric arteries or veins of 6 nude rats were inserted in central channels of porous hydroxyapatite cylinders and these pedicled grafts were implanted subcutaneously. One week later, the rats were perfused with ink-gelatin and euthanised and the femurs, tibias, and grafts were explanted. Samples were scanned using *μ*CT, decalcified, incubated with phosphotungstic acid (PTA) for contrast enhancement, rescanned, and processed histologically.

**Results:**

Contrast-enhanced *μ*CT displayed the course and branching of native bone vessels. Histologically, both central (−17%) and epiphyseal vessels (−58%) appeared smaller than in *μ*CT scans. Hydroxyapatite cylinders were thoroughly vascularised but did not display bone formation. Grafts with a central artery had more (+58%) and smaller (−52%) vessel branches compared to grafts with a vein.

**Conclusions:**

We present a relatively inexpensive and easy-to-perform two-step method to analyse bone and vessels by *μ*CT, suitable to assess a variety of bone-regenerative strategies.

## 1. Introduction

Bone formation and resorption depend on vessels to supply osteoblast and osteoclast progenitors, oxygen, and nutrients and guide bone formation by direct interaction with mature osteoblasts and osteoclasts [1]. Low blood supply or disturbed interaction between vessels and bone-forming cells leads to pathological conditions such as avascular bone necrosis (AVN) or fracture nonunion, in which bone does not heal after a fracture. In these cases, the required bone reconstruction is based on the use of autologous bone grafts, which generate additional morbidity and are limited by low availability [2, 3]. Bone substitute materials with enhanced osteoinductive capacity through the addition of progenitor/stem cells [4, 5] are being developed. However, these approaches have not yet proven efficient enough for a routine clinical use [6].

To test whether new therapies are able to reanimate ischemic bone, for example, by implanting engineered prevascularised bone tissue, bone vascularisation needs to be assessed. Bone vessel quantification, analysis of spatial relationships between vessels and bone trabeculae, and calculation of blood flow are nontrivial. Intravital observations such as two-photon microscopy are limited in depth and resolution and need a very thin bone cortex and sedation of animals [7]. Conversely, histological processing of explants is destructive and artefact-prone and does not allow analysis of the full vascular tree. Developed in recent years, the 3D reconstruction of vessels in explants using contrast-enhanced X-ray microtomography (*μ*CT) [8] has been described as a promising approach to solve this problem.

Common X-ray opaque vascular casts such as Microfill(R) [9, 10] or barium sulfate [11], used to analyse vessels between 10 *μ*m [10] and 240 *μ*m [12] in diameter, may compromise the analysis of bone trabeculae by displaying a similar, possibly inhomogenous signal in *μ*CT and creating beam hardening or scattering artefacts [11]. Additionally, though standard histological processing including enzymatic, immunohistochemical, and immunofluorescent staining is possible using these methods, analysis of vessels may be compromised by cast-induced distortion of the vascular morphology [13].

To solve these issues, we propose a two-step method with separate analysis of bone (e.g., trabecular size and spacing) and the corresponding vascular tree (e.g., number and size of vessel branches, vessel length), similar to the described double scanning of bone and bone marrow using osmium tetraoxide [14]. As a first step, bones are prepared with a radiolucent soft vascular cast specifically developed for histological analysis of vessels and capillaries, consisting of Indian ink combined with gelatin (ink-gelatin) [15]. After a first scan, bones are then decalcified and incubated with a solution of phosphotungstic acid (PTA), which enhances gelatin X-ray contrast by binding to the contained collagens. PTA does not otherwise alter tissue colour or stiffness, yet it has a low penetration depth due to its ionic nature [16, 17]. Decalcification removes ions from the tissue, thus increasing PTA penetration.

In this study we tested the applicability of this method for vascularised bones (e.g., rat femur and tibia). In particular, we investigated the reliability of the *μ*CT-based analysis of vessels contrasted with ink-gelatin/PTA and compared it to the standard histological assessment. We investigated whether enzymatic staining, immunohistochemistry, and immunofluorescence were feasible on contrast-enhanced samples. Finally, we aimed to demonstrate the application of this method on simple engineered grafts consisting of vascularised porous hydroxyapatite, adapted from an avascular necrosis model used in rats [18, 19].

## 2. Materials and Methods

### 2.1. Animals

Six male nude rats (RNU, Charles River Laboratories, Sulzfeld, Germany), 2–4 months old and weighing 300–400 g, were used in accordance with Swiss and European animal welfare regulations (permit BS 2598 by the cantonal veterinary agency, Basel, Switzerland). The methodological assessment of the reliability of ink-gelatin/PTA as a contrast agent for both *μ*CT and histology was performed on both femurs and both tibias. Each femur and each tibia was considered a single statistical unit. To exemplify the application of ink-gelatin/PTA in a realistic setting, per animal, one hydroxyapatite scaffold with either a central vein or a central artery was implanted, as detailed below.

### 2.2. Implantation of Hydroxyapatite

For implantations of pedicled grafts, rats were given 0.05 mg/kg buprenorphine subcutaneously for analgesia and anaesthetised using 2.5% isoflurane in 1 l/min oxygen. They were placed supine and the left groin was disinfected using Octenisept (Schülke & Mayr GmbH, Germany). After scalpel incision, the superficial inferior epigastric artery and vein were identified and dissected free of connective tissue using an operating microscope and microsurgical tools. Vessels were ligated and cut distally but left attached proximally to ensure perfusion from the femoral vessels. Two groups of three animals each were formed and either only the artery or only the vein was pulled through the 1.25 mm central channel of a 1 × 1 cm porous hydroxyapatite cylinder (Engipore™, Finceramica Faenza, Faenza, Italy), which was wrapped in a semipermeable membrane (Biobrane, UDL Laboratories Inc., Rockford, IL, USA) to prevent additional vascularisation from outside. A subcutaneous pocket was created by blunt dissection and the vascularised graft was inserted without kinking the vessel. The wound was closed with subcutaneous and intracutaneous sutures. One rat from the artery group died after operation due to unknown causes and was not used for analysis. Rats were placed under infrared light and monitored until full recovery. They were subsequently transferred back to their cage, monitored twice daily, and given subcutaneous buprenorphine every 12 hours in the next 3 days.

### 2.3. Perfusion with Ink-Gelatin and Explantation of Bones and Graft

After 1 week, rats were anaesthetised using 2.5% isoflurane in 1 l/min oxygen and given 100 mg/kg ketamine (Ketasol 100, Dr. E. Graeub AG, Switzerland) and 10 mg/kg xylazine (Xylasol, Dr. E. Graeub AG) intraperitoneally. The abdominal aorta was distally cannulated with a 22-gauge needle and flushed with a heparinised saline solution (100 I.U./ml in 0.9% NaCl, B. Braun, Switzerland), before an ink-gelatin mixture was applied (5% w/v Gelatine-Gold 180 bloom, Carl Roth GmbH, Germany; 50% v/v Indian Ink, Lefranc & Bourgeois, France; 4% w/v D-Mannitol, Carl Roth GmbH; bidistilled water). After perfusion, rats were euthanised by heart explantation and exsanguination. The cadavers were cooled to 4°C for 2-3 hours for polymerisation of the ink-gelatin. Thereafter, the vascularised graft as well as both femurs and tibias was dissected free of surrounding tissue and incubated in 1.5% paraformaldehyde in phosphate-buffered solution (Sigma-Aldrich) overnight.

### 2.4. Microtomography without Contrast Enhancement

Femurs and tibias were scanned after fixation in paraformaldehyde. The *μ*CT scans were performed using a nanotom® m (phoenix|x-ray, GE Sensing & Inspection Technologies GmbH, Wunstorf, Germany) equipped with a 180 kV/15 W nanofocus X-ray source. A tungsten transmission target, an accelerating voltage of 70 kV, and a beam current of 260 *μ*A were used. To increase mean energy of the photon spectrum and consequently reduce beam hardening artefacts, a 0.5 mm aluminium filter was inserted between source and specimen. A region of air was designated in all scans as suggested by the software (GE Sensing & Inspection Technologies GmbH) to standardize the grey-scale for data interpretation. 1440 equiangular projection images were acquired over 360° with an exposure time of 1 second. The radiographs were reconstructed using a cone beam filtered back-projection algorithm with the manufacturer's software phoenix datos|x 2.0.1 RTM (GE Sensing & Inspection Technologies GmbH). Whole bones were scanned and reconstructed with a voxel size of 18.5 *μ*m. Datasets were visualised using VG Studio MAX 2.1 (Volume Graphics GmbH, Heidelberg, Germany) and analysed using ImageJ with the BoneJ and 3D Shape plugins [20–22].

### 2.5. Decalcification

Bones and engineered grafts were decalcified by incubation in 15% EDTA pH 7.0 (Sigma-Aldrich) at 37°C. The decalcification solution was changed daily until samples were floating and soft when tested manually with a forceps. Engineered grafts were decalcified in 4 days, whereas bones required at least 14 days.

### 2.6. Contrast Enhancement Using Phosphotungstic Acid

5% w/v phosphotungstic acid (Sigma-Aldrich) in bidistilled water was prepared freshly before each use. Decalcified samples were completely immersed in this solution and incubated at room temperature for 24 (grafts) or 48 hours (bones). A longer incubation time ensured diffusion of the contrasting agent into the bone marrow, as indicated by preliminary scans using bones incubated for 24 hours.

### 2.7. Microtomography with Contrast Enhancement

Whole femurs and tibias were scanned after contrast enhancement as described above. Femoral heads and tibia plateaus were then separated. Heads, plateaus, and grafts were scanned as described above and reconstructed at a resolution of 2 *μ*m (heads, plateaus) or 7 *μ*m (grafts) per voxel. Manual analysis of the 3D datasets was performed using VG Studio MAX 2.1. Main blood vessel diameter and length within the bone shaft, the smallest distance between cortical bone and the center of the main vessel, and the number and diameter of branches from the main vessel were measured. In femoral heads and tibia plateaus, the number and diameter of vessels and their smallest distance to bone trabeculae were analysed. In grafts, the diameter of the central vessel and the number of vessels branching from the central vessel were analysed. All measurements were performed manually in 3D and on 3 orthogonal 2D sections, in analogy to histological analysis.

### 2.8. Histological Processing

After scanning, the decalcified and contrast-enhanced samples were dehydrated and embedded in paraffin. Femoral heads and tibia plateaus were embedded separately. Sections of 7–10 *μ*m thickness were stained with standard haematoxylin and eosin. Three representative midline sections were selected and vessel number, diameter, and distance to bone were analysed under a BX61 microscope (Olympus, Switzerland). For longitudinal sections of large vessels in whole bones, the largest diameter was identified along the vessel axis, the vessel center was defined as a point halfway across the diameter, and the distance from that point to the closest bone was measured. For cross-sections of small vessels, the largest and smallest diameters were identified and the center was defined as the crossing point of both diameters. The largest diameter and the distance from the center to the nearest bone were measured.

Staining for tartrate-resistant acid phosphatase (TRAP), as an exemplary enzymatic staining, was performed by incubation with a staining buffer (0.2 M sodium acetate 50 mM L+ tartaric acid, 1 mg/ml naphtol AS-MX phosphate, 1 mg/ml fast red TR salt, pH 5.0, all from Sigma-Aldrich) for 1 hour at 37°C.

For immunohistochemistry, primary antibodies against osterix (ab22552, Abcam UK, 1 : 80, no antigen retrieval) and CD31 (ab32457, Abcam UK, 1 : 50, heat enhanced antigen retrieval using a pressure cooker) were used. Appropriate biotinylated secondary antibodies were used (Abcam) and the staining was developed using the Vectastain ABC and Vectastain Fast Red kits (Vector Laboratories, UK).

For immunofluorescence, a primary Alexa488-labelled antibody against rat CD68 (ED1, AbDSerotec, 1 : 500, antigen retrieval with proteinase K) was used. Sections were counterstained with DAPI (Sigma-Aldrich) and analysed under fluorescence microscopy.

### 2.9. Data Analysis

Aggregation and analysis of the data with *t*-tests (comparisons of *μ*CT and histology) and Mann–Whitney *U* tests (comparisons of grafts with artery or vein) were performed with Microsoft Excel v. 14.6.2 and GraphPad Prism v.2 (GraphPad Software Inc., California, USA). A significance level *α* of 0.05 was set. Averages and standard deviations are presented.

## 3. Results

### 3.1. Analysis of Bone Architecture of the Rat Femur and Tibia by Microtomography

**Figure 1:**
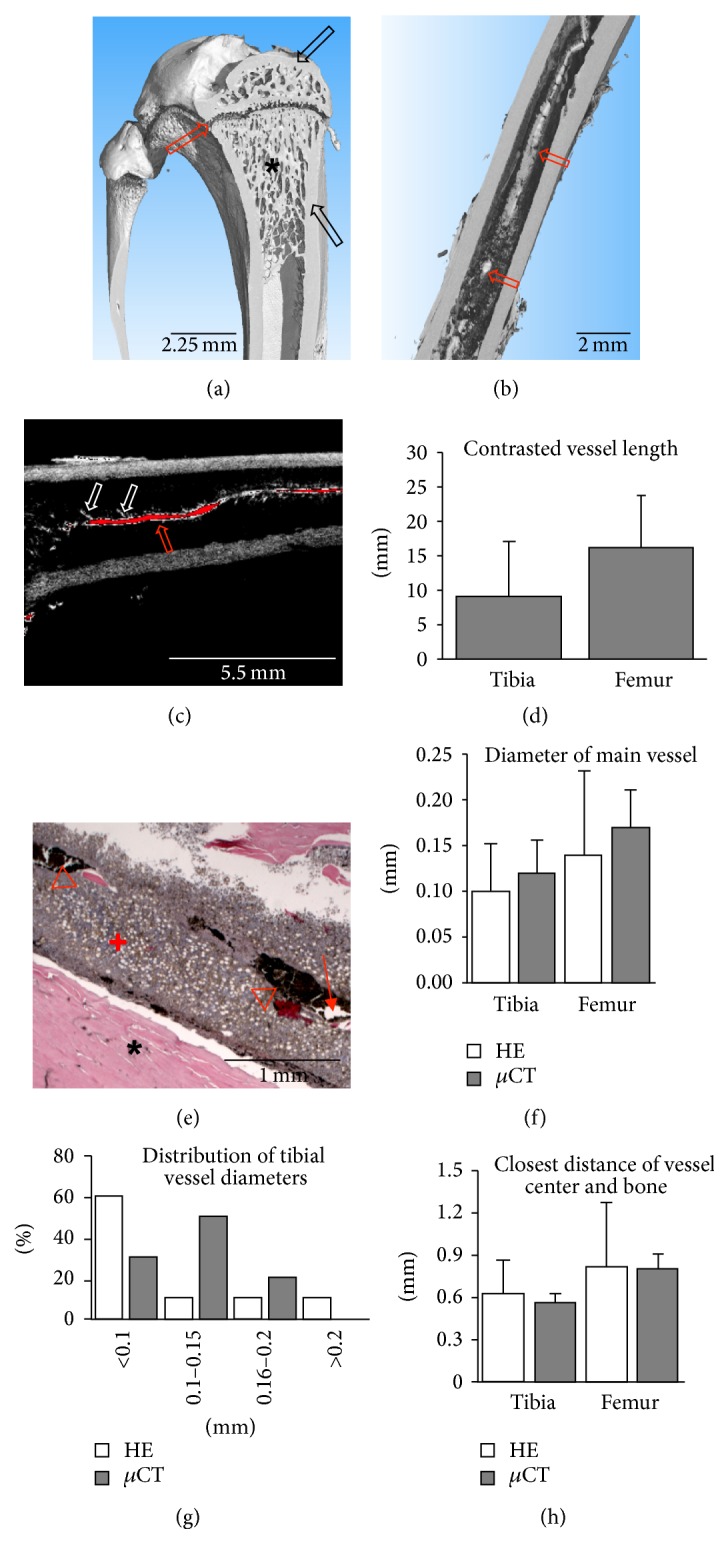
Bone architecture and contrast-enhanced vessel visualisation in rat long bones. (a) Bone architecture of rat tibia (3D rendering) with compact bone (black arrows), trabecular structure (star), and epiphyseal gap (red arrow). (b) 3D rendering of contrast-enhanced scan showing diaphysis of a rat femur with contrasted central vessel (red arrows). (c) 2D slice of the data set shown in (b). Red pixels represent grey values typical for vessels. Vessel course (red arrow) and branching (white arrows) are clearly visible. (d) Length of contrasted central vessel. (e) Haematoxylin and eosin staining of mid-diaphysis of a rat femur showing bone (star), bone marrow (plus), ink-gelatin-filled (triangles), and empty (arrow) vessels. (f) Diameter of central vessel measured in histology and microtomography. (g) Histogram of tibial vessel diameters (*n* = 10) showing a unimodal distribution, which is skewed left and wider for data from histology. Femoral vessel diameters display a similar distribution. (h) Closest distance of vessel center and bone measured in histology and microtomography.

Explanted femurs and tibias were scanned as described and reconstructed volumes did not display any artefacts, in particular no beam hardening or increased scattering (Figure 1(a)). Average bone length and mid-diaphyseal diameter were 38.8 ± 2.1 mm and 4.3 ± 0.5 mm for femurs and 36.5 ± 1.5 mm and 3.2 ± 0.4 mm for tibias. Structural analysis was performed on the reconstructed volumes by virtually extracting 1 mm^3^ of the trabecular structure of the proximal metaphysis, the distal metaphysis, and the proximal epiphysis. To illustrate bone parameters unique to *μ*CT analysis, the extracted volumes were then analysed for bone volume fraction (BV/TV), trabecular thickness (Tb.Th.), and trabecular spacing (Tb.Sp.). Results are displayed in Table 1 as averages and standard deviations of 10 samples.

### 3.2. Analysis of Rat Femurs and Tibias after Decalcification and Contrast Enhancement

After application of PTA for 48 hours, contrast enhancement of both the decalcified bone matrix and the central vessels inside the bone marrow cavity could be observed in reconstructed volumes (Figure 1(b)). Vessels displayed higher average grey values compared to bone matrix (184 ± 15 versus 129 ± 6, *P* = 0.002). The vessel course could be followed and branching points analysed manually by inspection of the reconstructed volumes (Figure 1(c)). An average of 16.3 ± 7.5 mm (femur) or 9.1 ± 8.1 mm (tibia) of the central vessel could be visualised, representing, respectively, 44% and 23% of the total bone length (Figure 1(d)). Similar numbers of branching vessels with a similar average diameter were found in femurs (14 ± 10 branches, 0.06 ± 0.007 mm diameter) and tibias (14 ± 8 branches, 0.06 ± 0.009 mm diameter). Although histological processing of samples was straightforward, vessel analysis required selection of representative sections. It was possible to analyse vessel diameter and distance to bone. Vessels appeared mostly as longitudinal sections of large vessels or cross-sections of small vessels with an elliptical shape (Figure 1(e)). Due to the 2D nature of standard histological slides, the uncontrollable wasting of sections due to cutting errors and the disproportionate labour required to produce serial cross-sections, vessel course, or branching were not measured. Interestingly, ink-gelatin contrast was not homogenously present in central vessels, which was in line with the incomplete visualisation of vessels by *μ*CT. The vessel diameter and closest distance of the vessel center to the corticalis were the only quantitative measurements that could be compared between histology and *μ*CT. Both did not show any significant difference (Figures 1(f), 1(g), and 1(h)), though vessel diameter appeared smaller on average (−17%) and more variable in histology compared to *μ*CT, possibly as a result of dehydration and shrinking during histological processing or sampling issues due to the use of longitudinal sections.

### 3.3. Analysis of Femoral Heads and Tibia Plateaus after Decalcification and Contrast Enhancement

**Figure 2:**
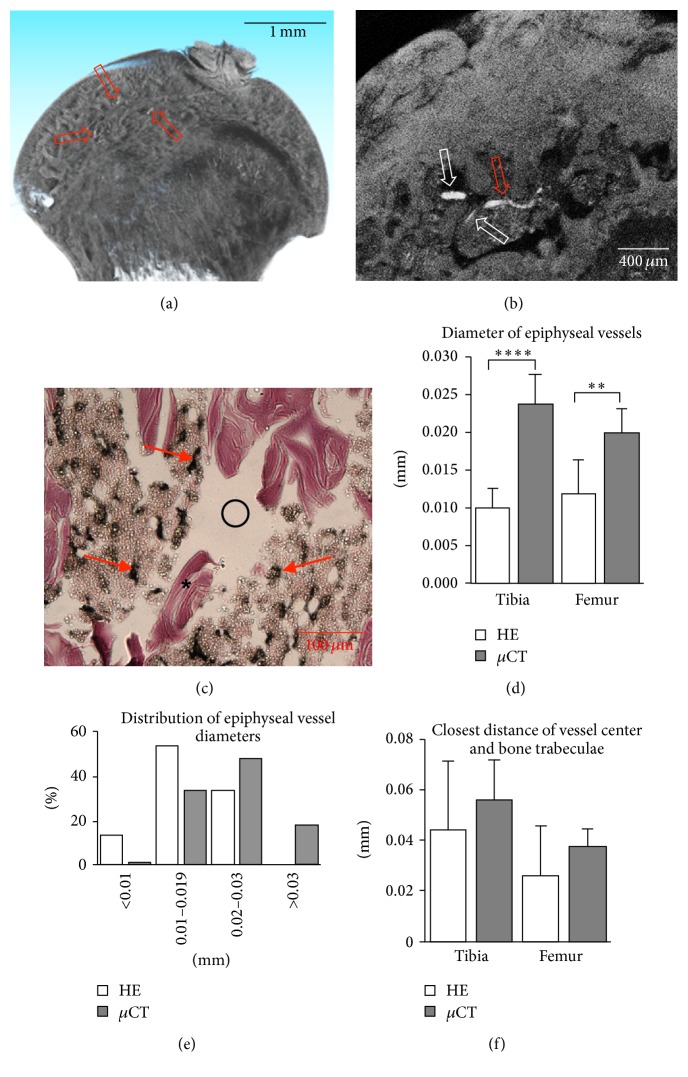
Analysis of small epiphyseal vessels in femoral heads and tibia plateaus. 3D rendering (a) and 2D slice (b) of contrast-enhanced scans showing contrasted vessels (red arrows) and vessel branching (white arrows). (c) H&E staining showing ink-gelatin filled vessels (arrows) and bone trabeculae (star). Large cutting artefacts are visible (circle). (d) Diameter of the epiphyseal vessels measured by histology and microtomography (^*∗∗*^*P* < 0.01, ^*∗∗∗∗*^*P* < 0.00001). (e) Illustrative histogram of vessel diameters (*n* = 113) of a single femoral head showing a unimodal distribution, which is shifted left for histological data. (f) Closest distance of vessel center and bone trabeculae measured by histology and microtomography.

High-resolution scans were performed on separated femoral heads and tibia plateaus to analyse small-calibre (approximately 10 *μ*m to 30 *μ*m diameter) vessels. Volume reconstructions were performed with 2 *μ*m isometric voxels. Vessels were distinguished in reconstructed volumes both with 3D visualisation and with 2D visualisation of serial sections (Figures 2(a) and 2(b)). Manual analysis of the reconstructed volumes revealed the course and branching of vessels. Conversely, histological sections of the same samples suffered cutting artefacts and required selection of the best sections for vessel analysis (Figure 2(c)). In both femoral heads and tibia plateaus, vessels appeared on average 58% smaller in diameter in histology as compared to *μ*CT (Figures 2(d) and 2(e)), possibly as a result of dehydration and shrinking during histological processing. However, the closest distance from the vessel center to the bone trabeculae was not significantly different (Figure 2(f)).

### 3.4. Enzymatic, Immunohistochemical, and Immunofluorescent Staining

**Figure 3:**
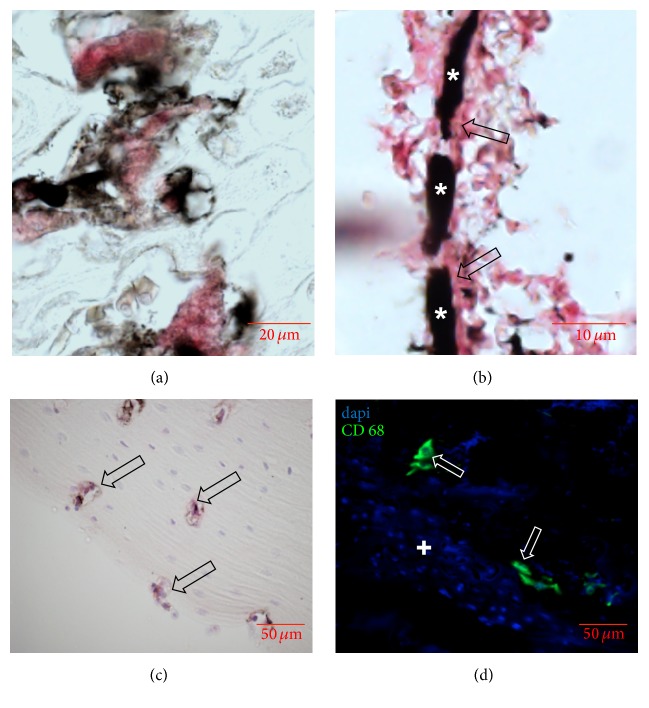
Enzymatic, immunohistochemical, and immunofluorescent staining on sections of femurs and tibias after contrast enhancement. (a) TRAP staining showing multinucleated osteoclasts (red) in the epiphyseal gap. (b) CD31 staining showing a small ink-gelatin contrasted vessel (star) with positively stained endothelial lining (arrows). (c) Immunohistochemistry for osteoblasts (arrow) in the corticalis. (d) Immunofluorescence for CD68 showing single rat macrophages (arrows) inside the bone marrow (plus).

Although standard haematoxylin and eosin staining was feasible, we could not exclude potential artefacts introduced by the contrast agent with more elaborate staining. Therefore we performed series of enzymatic, immunohistological, and immunofluorescent staining on sections of femurs and tibias. TRAP staining for osteoclasts (Figure 3(a)), CD31 staining for endothelial cells (Figure 3(b)), osterix staining for osteoblasts (Figure 3(c)), and CD68 immunofluorescence for macrophages (Figure 3(d)) were feasible in all samples.

### 3.5. Analysis of Vascularised Porous Hydroxyapatite after Decalcification and Contrast Enhancement

**Figure 4:**
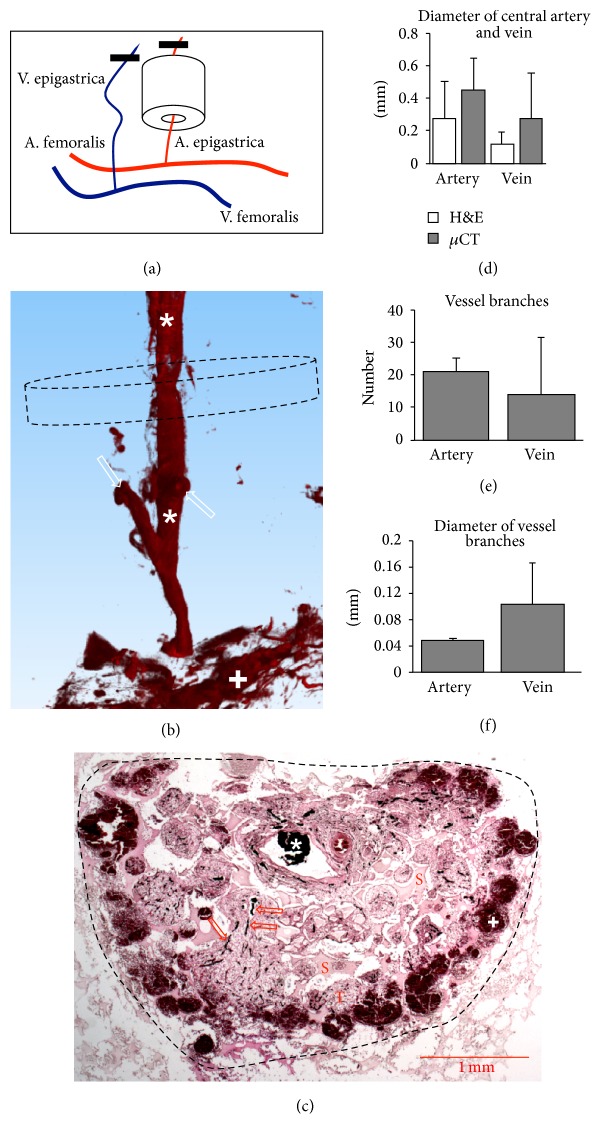
Analysis of vascularised porous hydroxyapatite. (a) Schematic of implantation. Superficial inferior epigastric vessels were identified, ligated (black bar), and cut distally. Either the artery (as shown) or the vein was pulled through the hydroxyapatite cylinder. (b) 3D rendering of contrasted central vein (star) with branches (white arrows), some diffuse contrast enhancement in the periphery (plus). After decalcification, the Engipore cylinder is no longer visible. Dashed line represents circular cross-section of Engipore cylinder. (c) H&E staining showing ink-gelatin contrasted central vein (star), small branching vessels (arrows), scaffold material (S) and pores filled with connective tissue (T), and peripheral haemorrhage with ink-gelatin (plus). Dashed line represents circular cross-section of Engipore cylinder. (d) Diameter of central artery and vein measured by histology and microtomography. (e) Number of vessels branching from central artery or vein. (f) Diameter of vessels branching from central artery or vein.

In order to demonstrate an exemplifying application of our method, we engineered a simple vascularised graft (Figure 4(a)). One week after implantation, we analysed tissue formation and vascularisation. *μ*CT analysis after contrast enhancement and reconstruction with 7 *μ*m isometric voxels clearly evidenced a perfused central vessel with branches (Figure 4(b)). Diffuse contrast enhancement at the periphery of the constructs indicated that ink-gelatin had spilled into the tissue. Indeed, upon histological examination, the perfusion of the central vessel, smaller vessels throughout the tissue, and a peripheral haemorrhage were visible (Figure 4(c)). Quantification of vessel diameter, number, and diameter of branches showed no significant difference (Figures 4(d), 4(e), and 4(f)), although constructs with central arteries tended to display larger diameter (+60%), more branches (+58%), and smaller branch diameters (−52%) compared to constructs with central veins. The parameters were not significantly different compared to vascularisation in rat femurs and tibias.

## 4. Discussion

In this study we demonstrated the use of ink-gelatin and PTA as contrast enhancement for *μ*CT analysis of bone vasculature. We found that scanning after decalcification and contrast enhancement allowed for an evaluation of the course, branching, and average diameter of vessels, as well as the closest distance from the vessel center to adjacent bone with both large (150 *μ*m) and small (10 *μ*m–30 *μ*m) vessels. Moreover, the method did not interfere with standard histological processing, enzymatic staining, immunohistochemistry, and immunofluorescence on the same samples.

Considering the significant limitations of X-ray opaque vascular casting, such as irreversible contrasting of vessels before bone scans, introduction of beam hardening and scattering artefacts, or difficulty of histological processing thereafter [8–11, 13, 23, 24], we aimed to provide an alternative method with a potentially higher precision and simplified handling. We identified a combination of two existing methods for *μ*CT and histological contrast enhancement. On one hand, the infiltration of vessels using Indian ink and gelatin is an accepted method to analyse blood flow through vessels in histological sections [15]. The ink-gelatin is applied to the circulation and left to polymerise after euthanasia, providing a soft and coloured vascular cast. On the other hand, contrast enhancement of soft tissues in *μ*CT has been proposed with a variety of contrasting agents [16, 17]. Interestingly, only PTA has demonstrated selectivity for collagens [25], without significantly altering the stiffness or colour of the tissue [26]. Although direct perfusion with PTA may contrast vessels in soft tissue thanks to its limited penetration in a short time [27], the resulting contrast is insufficient in bone and may be adversely affected by decalcification.

Sequential contrast enhancement ensures that bone can be analysed by standard *μ*CT procedures [28, 29] while avoiding the generation of artefacts or difficult image segmentation commonly encountered with vascular casting. Indeed, by using this method, vessel contrasting becomes one of several optional analyses after explantation of the ink-gelatin-perfused sample.

Although many types of vessel analyses on 3D reconstructed data have been previously proposed, such as automated counting of vascular diameters, branching, or analysis of tortuosity, we chose manual measurement of diameters, number of branches from the central vessels, and distance of the vessel center to the adjacent bone trabeculae. These measurements indeed share a straightforward interpretation [11, 30] and avoid artefacts commonly generated by some automated algorithms [31–34]. Although still requiring occasional manual corrections, newer highly automated algorithms with greater reliability [12, 35] should be applied when available in future applications of the herein described method to add further information and facilitate data collection.

During both *μ*CT and histological analysis, we observed an incomplete filling of the central bone vessels with contrasting agent as well as peripheral haemorrhages from ruptured vessels, suggesting that the optimal flow properties and the optimal application of ink-gelatin still need to be characterised for future studies. Nevertheless, PTA was able to penetrate a whole rat femur/tibia and contrast the central vessel by diffusion, presenting a very useful property for the analysis of larger grafts and grafts with a closed bone marrow space.

The analysis of a simple, vascularised graft using porous hydroxyapatite with a central vein or artery exemplified an application of our method. Based on the limited data presented here, it appeared that the use of a single vein or a single artery had no impact on graft vascularisation. Although our concrete example may not be meaningful in a broader context of bone vascularisation, our method allowed a comparison of vascular branches and average branch diameter, both crucial aspects for tissue perfusion. For a more thorough evaluation of graft vascularisation, dedicated studies with greater statistical power are required, featuring previously published approaches such as vascular bundles of artery and vein [18, 36], vascular loops [15, 37], or wrapping with well-vascularised muscle tissue [38]. Though granulation tissue with rudimentary vasculature, sufficient for demonstration purposes in this study, formed after one week, longer in vivo incubation may be necessary for full maturation of the vascular tree.

In conclusion,* this study provides a relatively inexpensive and easy-to-perform two-step technique based on the use of ink-gelatin and PTA for the analysis of bone and vessels by μCT*. This technique could be applied to answer a variety of different questions in bone tissue engineering.

## Figures and Tables

**Table 1:** Bone volume fraction (BV/TV, no unit), trabecular thickness (Tb.Th., in mm), and trabecular spacing (Tb.Sp., in mm) of 1 mm^3^ of the proximal metaphysis, the distal metaphysis, and the proximal epiphysis of rat femurs and tibias. Averages and standard deviations of 10 samples are shown.

			Femur	SD	Tibia	SD
BV/TV (no unit)	*Proximal *	*Epiphysis*	0.448	*0.104*	0.286	*1,000*
		*Metaphysis*	0.386	*0.092*	0.358	*0.12*
	*Distal*		0.481	*0.38*	0.127	*0.13*

Tb.Th. mm	*Proximal *	*Epiphysis*	0.026	*0.017*	0.009	*0.001*
		*Metaphysis*	0.159	*0.039*	0.098	*0.027*
	*Distal*		0.151	*0.143*	0.041	*0.048*

Tb.Sp. mm	*Proximal *	*Epiphysis*	0.064	*0.027*	0.026	*0.001*
		*Metaphysis*	0.309	*0.08*	0.208	*0.067*
	*Distal*		0.233	*0.344*	0.023	*0.178*

## Additional Points

*Key Points.* (i) Bone vessels can be visualised using ink-gelatin and phosphotungstic acid. (ii) The size, branching, and distance from bone can be analysed. (iii) This method can be used to evaluate regenerative strategies in bone.
